# Supplementary material for: Comprehensive Insight into Chemical Stability of Important Antidiabetic Drug Vildagliptin Using Chromatography (LC-UV and UHPLC-DAD-MS) and Spectroscopy (Mid-IR and NIR with PCA)
Source: Molecules. 2021 Sep 16;26(18):5632. doi: 10.3390/molecules26185632 (PMC8472283; doi:10.3390/molecules26185632)
Supplement: Supplementary file 1 [file molecules-26-05632-s001.zip › molecules-1374854-supplementary.pdf]

Material and Methods

Validation of Quantitative LC-UV Method

Robustness study was performed by deliberately changing some chromatographic conditions. The flow rate of the mobile phase was changed over the range of 1.0-1.4 mL/min. The content of acetonitrile in the mobile phase varied from 18 to 22% while the detection wavelength was changed in the range 207-213 nm. One factor was changed at one time to estimate the differences in the peak areas, retention time (t_R) of vildagliptin and I.S., as well as, in resolution (R_S) between the peaks of interest.

Stock Solutions

Stock solutions of vildagliptin and I.S. were prepared by dissolving both substances in methanol to obtain the concentrations of 1 mg/mL. These stock solutions were stored in the dark at 4°C and were found to be stable for several weeks.

Calibration

Working solutions of vildagliptin were prepared by dispensing 0.4-1.9 mL volumes from the stock solution to 10 mL volumetric flasks, to reach the concentration range 40-190 μg/mL. To each flask, 0.4 mL of the I.S. stock solution was added. After adjusting with methanol to the mark, each sample was injected onto the column. These calibration procedures were repeated six times. For each calibration set, the ratio of peak areas (vildagliptin/I.S.) was plotted against the corresponding concentration of vildagliptin, to construct the calibration equation by the least-squares method. The limit of detection (LOD) and the limit of quantification (LOQ) were determined from the SD of the intercept and the slope of the mean regression line.

Precision and Accuracy

For the precision study, the working solutions of vildagliptin were prepared by dispensing 0.5, 1.1 and 1.8 mL volumes from the stock solution to 10 mL volumetric flasks, to reach the concentrations 50, 110 and 180 μg/mL. To each flask, 0.4 mL of the I.S. stock solution was added. After adjusting with methanol to the mark, the injections from each working solution were made onto the column three times a day. For each concentration, three repetitions were made in three subsequent days. The precision of the method was calculated as relative standard deviations (RSD) for these intra-day and inter-day results.

Accuracy was estimated by determining vildagliptin in six accurately weighed samples of powdered Galvus® tablets. The portions of powdered tablets equivalent to ca. 50 mg of vildagliptin were transferred to 25 mL volumetric flasks, mixed with 15 mL of methanol, sonicated for 30 min, diluted to the mark and filtered by nylon membrane filters (0.45 μm). Then, 1.0 mL of each solution was dispensed to 10 mL volumetric flasks, mixed with 0.40 mL volumes of the I.S. stock solution, diluted to the mark with methanol and injected onto the column. The obtained concentrations of vildagliptin were calculated using the mean calibration equation, compared with the declared values and expressed as percentage recoveries.

Results and Discussion

Optimization and Validation of LC-UV Method for Quantitative Measurements of Vildagliptin

The LC-UV method was optimized to separate vildagliptin and I.S. (phenacetin), as well as, the possible related compounds obtained from forced degradation. It was found that simple mobile phase containing 2 mM ammonium acetate and acetonitrile at 80:20 (v/v) ratio was sufficiently effective for separation of the peaks of interest in a reasonable time, as well as, for reduction of the peak tailing. The chromatograms showed that the peaks of vildagliptin were free from interferences of these from degradation products (Figure S1). Moreover, the chromatograms obtained for the samples from powdered tablets containing vildagliptin showed that the peaks of interest were free from interferences from excipients (data not presented). Thus, the selectivity of the method was proved.

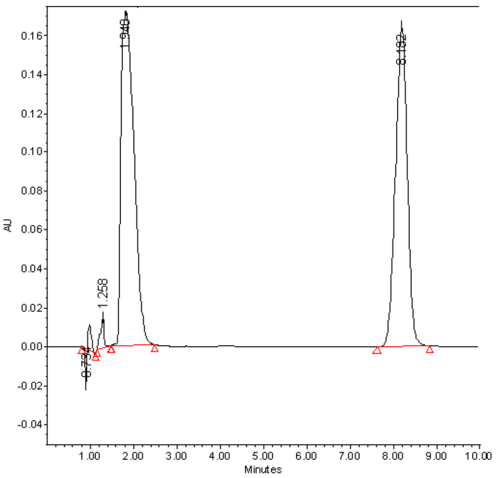

A

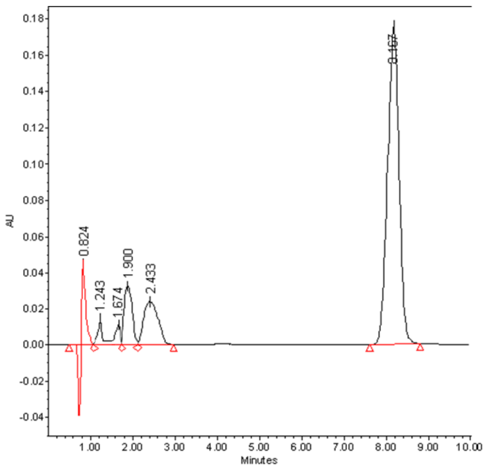

B

Figure S1. Chromatograms from our LC-UV method: A) vildagliptin (t_R=1.948 min) and I.S. (8.182 min) in the calibration solution; B) vildagliptin and I.S. in the presence of vildagliptin degradation products after degradation in alkaline medium at 23°C; (t_R=retention time).

Robustness

Robustness of the method was checked by comparing the retention times (t_R), peak areas and resolution (R_S) between the peaks of vildagliptin and I.S. under slightly changed analytical parameters, *i.e.* the flow rate of the mobile phase (1.2±0.2 mL/min), acetonitrile content in the mobile phase (20±2%, v/v) and the detection wavelength (210±3 nm). When small changes of the flow rate of the mobile phase and detection wavelength were introduced, uniformity of the peak areas, t_R values and R_S was observed, confirming the robustness of the method. The changes in percentage content of acetonitrile in the mobile phase, however, slightly affected the t_R values and resolution between the peaks of interest (Table S1).

Table S1. Results for the robustness study of our LC-UV method for the determination of vildagliptin (V); (n=3).

Parameter		Peak area		t_R [min]		R_S
		V	I.S.	V	I.S.	
Wavelength [nm]	207	1240596	1966542	1.923	8.203	7.55
	210	1253350	1989901	1.915	8.167	7.52
	213	1248689	1963010	1.924	8.165	7.55
Flow rate of the mobile phase [mL/min]	1.0	1256989	1957904	1.926	8.046	7.59
	1.2	1253350	1989901	1.915	8.167	7.52
	1.4	1253989	1969293	1.884	8.007	7.54
Acetonitrile in the mobile phase [%, v/v]	18	1258275	1958845	2.312	7.939	6.96
	20	1253350	1989901	1.915	8.167	7.52
	22	1258875	1949472	1.589	8.547	7.71

t_R=retention time; R_S=resolution.

Linearity

The linearity of detector response to different concentrations of vildagliptin was confirmed in the range 40-190 μg/mL, giving the mean (±SD) regression equation y = 0.00618 ± 0.000075x + 0.02613 ± 0.005617, where y was the peak area ratio (vildagliptin/I.S.) and x was the concentration of vildagliptin in μg/mL. The mean (±SD) determination coefficient (R^2) for six calibration sets was 0.9997 ± 0.00013. The LOD and LOQ were calculated from the

standard deviation of the intercept and slope of the regression line and amounted 2.99 μg/mL and 9.09 μg/mL, respectively.

Precision and Accuracy

Precision of the method was determined by analyzing three different concentrations of vildagliptin on the same day and repeating the analysis on three subsequent days. The RSD values for these intra-day determinations were obtained in the range 0.55-0.26% (n=3) while for inter-day they were in the range 1.46-0.64% (n=9). Accuracy of the method was estimated by the determination of vildagliptin in six samples of powdered tablets and comparison of the determined amounts of the drug to the declared values (n=6). The mean (±SD) recovery of vildagliptin from tablets was obtained as 99.86±1.36%. Thus, the precision and accuracy of the proposed LC-UV method were proved.

Identification of Vildagliptin Degradation Products by UHPLC-DAD-MS

There are some other minor peaks at our BPC chromatograms of the analyzed samples, namely Impurities 1-3 (Imp 1-3), probably from impurities present in vildagliptin pure substance. Their MS and UV/VIS spectra are shown in Figures S2-S4.

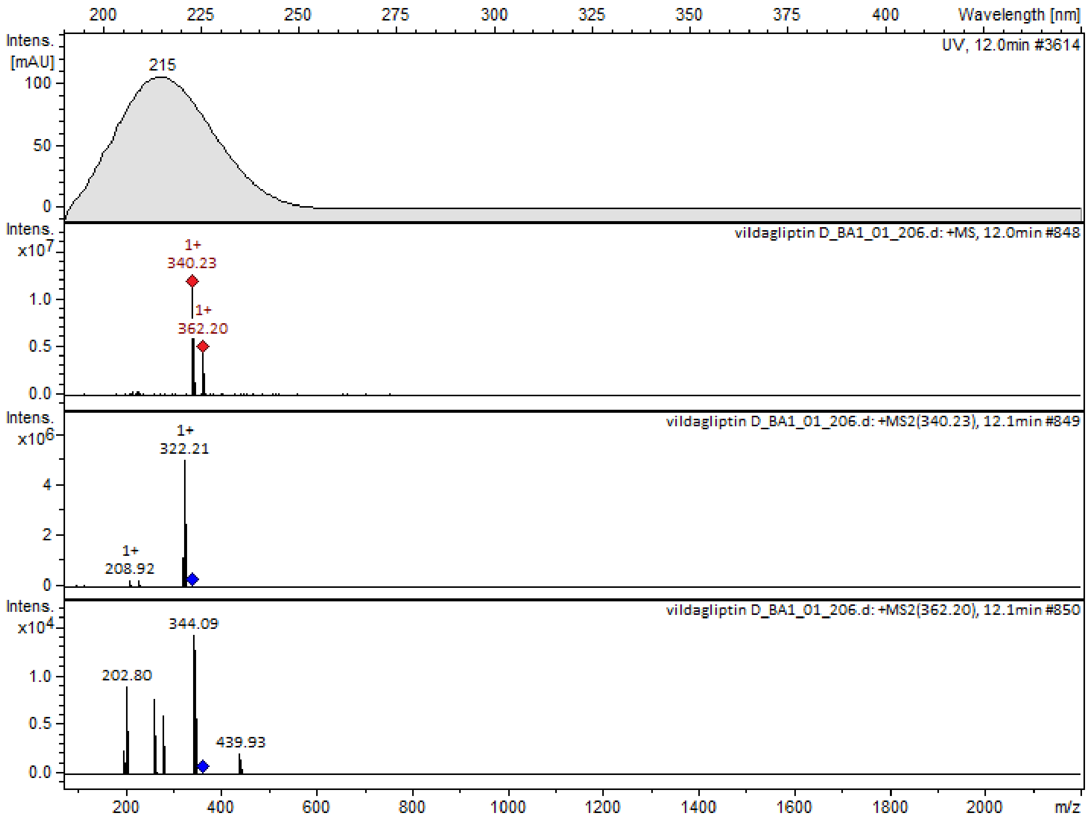

Figure S2. MS and UV/VIS spectra of Imp 1.

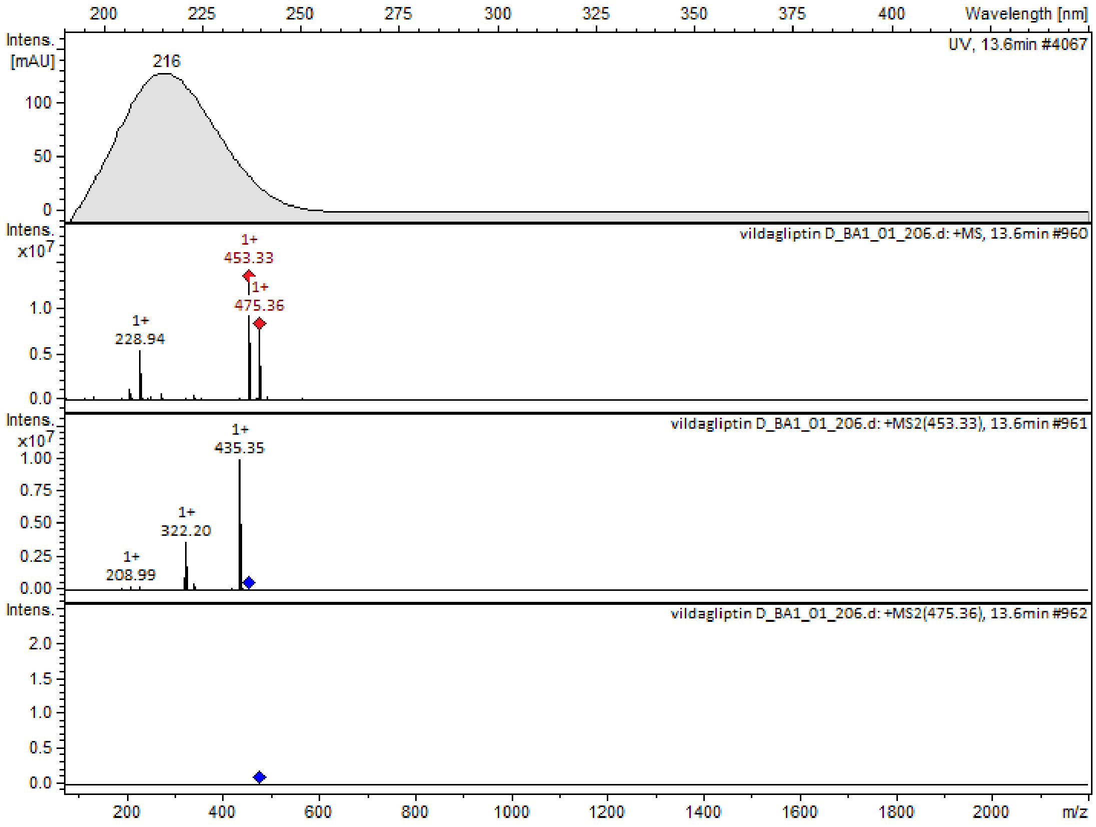

Figure S3. MS and UV/VIS spectra of Imp 2.

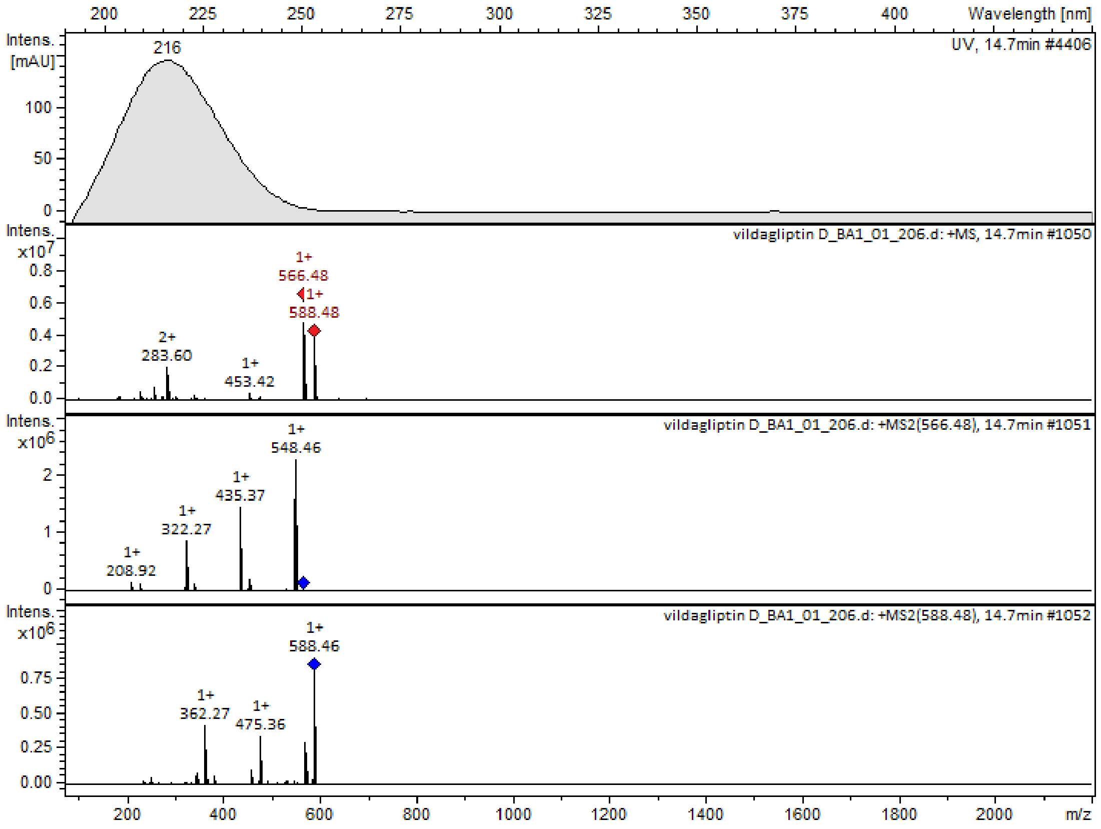

Figure S4. MS and UV/VIS spectra of Imp 3.
